# Evaluation of Food Retail Policies Implementation in China Using the Healthy Food Environment Policy Index

**DOI:** 10.3390/nu17172815

**Published:** 2025-08-29

**Authors:** Shuyi Zhou, Na Zhang, Zhenhui Li, Wenli Zhu, Suying Chang, Ali Shirazi, Shijie Gao, Yurong Xi, Yujie Fang, Man Zhang, Guansheng Ma

**Affiliations:** 1 School of Public Health, Peking University, Beijing 100191, China; shuyizhou78@163.com (S.Z.); zhangna@bjmu.edu.cn (N.Z.); lizhenhui@bjmu.edu.cn (Z.L.); zhuwenli@bjmu.edu.cn (W.Z.); worldGao2@163.com (S.G.); fangyujie@pku.edu.cn (Y.F.); 2 Laboratory of Toxicological Research and Risk Assessment for Food Safety, Peking University, Beijing 100191, China; 3 Health and Nutrition Section, UNICEF Office for China, 12 Sanlitun Lu, Chaoyang District, Beijing 100600, China; schang@unicef.org (S.C.); hdalvi@unicef.org (A.S.); 4 School of Global Public Health, New York University, New York, NY 10003, USA; yurongxi515@gmail.com; 5 Institute of Food and Nutrition Development, Ministry of Agriculture and Rural Affairs, Beijing 100000, China

**Keywords:** healthy food environment policy index, food retail, policy implementation, China, food environment, public health policy

## Abstract

**Objectives**: Despite the importance of food environments in shaping dietary behaviors and diet-related noncommunicable diseases, no standardized and validated method has been used to assess this policy domain in China. This study aimed to benchmark China’s food retail policies against international benchmarking to identify implementation gaps and propose evidence-based strategies for improving food environments. **Methods**: Using the standardized and validated Healthy Food Environment Policy Index (Food-EPI), we assessed four food retail indicators: restrictive zoning for fast-food outlets (RETAIL 1), healthy food accessibility (RETAIL 2), institutional support systems (RETAIL 3), and food service promotion (RETAIL 4). A multidisciplinary expert panel (*n* = 13) from academia, public health, and industry conducted structured assessments using a standardized scoring tool (1–5). Scores were converted to implementation percentages and categorized into four levels. Descriptive statistics (frequencies, percentages, and mean ± SD) were summarized. **Results**: Sixteen food retail policies were analyzed, with 62.5% (*n* = 10) of provincial policies focusing on fast-food zoning and healthy food access, and 37.5% (*n* = 6) of national policies targeting government support and healthy food promotion. Regulations comprised 55% (*n* = 11), mainly addressing zoning and access, while guidelines accounted for 45% (*n* = 9), primarily promotional. Overall, the food retail domain was rated as low level (2.3 ± 1.1, 45.8% implementation). Among the four indicators, healthy food access in outlets (2.6 ± 1.3, 52.4%) and promotion of healthy food in services (2.5 ± 1.0, 50.8%) were at medium implementation levels. In contrast, local fast-food zoning restrictions (1.7 ± 0.6, 33.8%) and government support for healthy food (2.3 ± 1.1, 46.2%) remained at low levels. **Conclusions**: China’s food retail policies showed inconsistent implementation levels, with strong provincial execution in accessibility and promotion but weak national coordination in zoning and support systems. To align with the WHO and China’s goals, priorities are unified national frameworks, enforceable standards, equity-focused monitoring, and strengthened multi-sector collaboration.

## 1. Introduction

The food environment constitutes the multifaceted interplay of physical, economic, policy, and sociocultural factors that shape individuals’ access to, availability of, and choices regarding food and beverages, thereby influencing dietary patterns and health outcomes [1]. The food retail environment, as a part of the food environment, refers to the availability, type, and density of food outlets within a community, along with the diversity and nutritional quality of products offered and the pricing and promotion strategies that shape consumer access and choices [2]. Unhealthy food retail environments have been consistently linked to poor dietary behaviors and adverse health outcomes. A strong correlation has been identified between the density of unhealthy food outlets and increased body mass index (BMI) risk [3,4,5]. For instance, research from Beijing found a significant association between a higher density of convenience stores near schools and a 13% increased risk of obesity in school-aged children [6]. Accelerated urbanization between 2008 and 2020 led to a 27% surge in fast-food outlet density near schools in Belgium, paralleling a 15% increase in childhood obesity rates in affected regions [3,6]. China’s rapid retail modernization in the early 2000s exemplifies this trend: widespread proliferation of energy-dense processed foods contributed to a 4.97-unit BMI increase among children and a 3.63-fold elevated risk of overweight status [7].

Given these detrimental health impacts, it becomes imperative to consider effective interventions. Evidence-based policy interventions targeting the food retail environment, such as zoning restrictions on unhealthy outlets and subsidies for fresh produce, have demonstrated efficacy in improving dietary quality and reducing obesity-related morbidity [8,9]. However, the successful implementation and ultimate impact of such policies are often modulated by complex factors, including socioeconomic disparities, urbanization patterns, and prevailing regulatory frameworks. Food retail policies influence the food environment by modifying availability, accessibility, and promotion of food products, which in turn shape consumer dietary behaviors and ultimately impact noncommunicable diseases, such as obesity and CVDs [10]. Understanding these mediating factors is crucial for designing equitable and effective food systems, underscoring the pivotal role of well-tailored policy in shaping public health outcomes [9]. Recognizing the global burden of NCDs, the World Health Organization (WHO) has prioritized their prevention through the Global Action Plan for the Prevention and Control of NCDs 2013–2020 [11]. Complementing this, China’s Healthy China 2030 initiative highlights the improvement of food environments as a fundamental strategy to reduce diet-related NCDs and enhance population health [12].

Despite food retail environments being widely recognized as a social determinant of health, China lacks a systematic, policy-oriented evaluation of its own retail food environment. This analytical gap significantly impedes the development of targeted, evidence-based policy solutions, a concern that is particularly acute given the increasing prevalence of diet-related chronic disease. This analytical gap significantly impedes the development of targeted, evidence-based policy solutions. The absence of a standardized assessment framework makes it difficult for policymakers to effectively quantify the impact of existing policies, identify specific weaknesses and implementation gaps, and benchmark progress against national health targets or international best practices. This concern is particularly urgent given the increasing prevalence of diet-related chronic disease.

Healthy Food Environment Policy Index (Food-EPI) is a globally validated tool that was developed by INFORMAS (International Network for Food and Obesity/NCDs Research, Monitoring and Action Support) [13,14]. The framework consists of two components, policy and infrastructure support, which includes 13 domains and 47 good practice indicators. The policy component includes seven domains: food composition, labelling, promotion, provision, retail, pricing, and trade and investment. The infrastructure component includes six domains: leadership, governance, monitoring, funding, platforms for interaction, and health-in-all-policies. Each domain comprises specific indicators used to evaluate the extent of government implementation relative to international best practice [15]. Originally launched in 2012, Food-EPI has been applied in 58 countries, facilitating cross-national benchmarking and identifying best practices for food environment policies [14,16].

These studies have demonstrated the framework’s effectiveness in identifying policy strengths and weaknesses, as well as guiding evidence-based policy recommendations [17]. However, a major research gap remains, as few studies have applied the Food-EPI in China, and none have systematically assessed the food retail policy domain. This is largely due to limited local adaptation of the framework, insufficient standardized policy data, and the complexity of multi-level governance, which have hindered comprehensive evaluation and international benchmarking.

To address this research gap, this study applied the Food-EPI to assess China’s food retail policies. The aim of this study is to systematically evaluate the alignment of China’s food retail policies with international best practices using the Food-EPI framework. The innovation lies in providing the first comprehensive assessment of this kind in China, thereby providing valuable data for future research. This study offers global added value by demonstrating the applicability of the Food-EPI framework in a large, rapidly developing country with a unique policy context and by offering valuable policy recommendations to mitigate diet-related noncommunicable diseases and promote public health equity in China.

## 2. Materials and Methods

This study specifically focused on the food retail domain within the Food-EPI framework. The food retail domain consists of four key indicators originally established to represent critical policy levers for shaping consumer choices in the retail environment [15,18]: T Specifically, these four indicators are: restrictive zoning for fast-food outlets (RETAIL 1), healthy food accessibility in retail outlets (RETAIL 2), government support for healthy food initiatives (RETAIL 3), and promotion of healthy food in service settings (RETAIL 4) (Table 1). We fully adopted these four indicators to ensure that our evaluation was consistent with the internationally recognized methodology, enabling comparability with other countries.

The Food-EPI framework, while globally validated, was adapted to the Chinese context through a multi-step process, following the methodology developed by Li et al. in 2024 [19]. This process included a contextualization of the global indicators to reflect China’s specific food policy landscape. A national expert panel, as described in Section 2.3, reached a consensus on the relevance and interpretation of each indicator, thereby validating its use for assessing the food retail environment in China.

**Figure 1:**
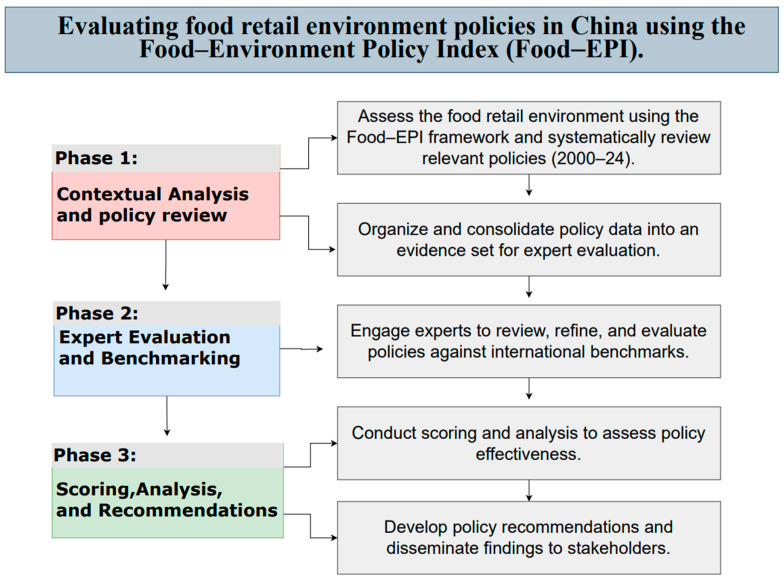
Evaluating food retail-related policies in China using the Food–EPI.

In brief, the implementation process included three key phases as depicted in Figure 1: (1) Contextual Analysis and Policy Review, which involved assessing the food retail environment and systematically reviewing relevant policies from 2000–2024; (2) Expert Evaluation and Benchmarking, where experts reviewed and evaluated policies against international benchmarks; and (3) Scoring, Analysis, and Recommendations, which focused on assessing policy effectiveness and developing recommendations.

### 2.1. Contextual Analysis and Policy Review

To ensure accuracy, the methodology and food retail indicators of Food-EPI were translated into Chinese. The translation was subsequently validated by other members of our research team who possess strong bilingual proficiency and relevant expertise in food policy evaluation [19]. A comprehensive review of policy documents from January 2000 to June 2024 was conducted, including laws, regulations, guidelines, standards, and action plans, with detailed definitions provided in our previous study [19]. The evidence collection utilized a structured, multi-tiered approach, including stakeholder mapping, systematic website searches, direct engagement with organizations, and targeted academic database searches [19].

### 2.2. Consolidation and Validation of Evidence

The collected data from a structured review of government policy documents, reports, and national health statistics were compiled into an evidence set. This set detailed policy names, release dates, issuing authorities, hierarchical levels, and relevant content. This evidence set underwent initial validation by ten Chinese experts with expertise in public health nutrition, food policy, and retail regulation, through two rounds of online consultations, ensuring its rigor and relevance [19].

### 2.3. Expert-Led Validation and Refinement of the Evidence Set

For the subsequent evaluation of China’s food retail policies using the Food-EPI framework, this expert panel was expanded to a total of 13 experts, including the initial ten validators and three additional experts, who collectively evaluated the policies. The experts were strategically selected to ensure representation from diverse professional domains crucial to the food retail environment, such as nutrition, public health, and the food industry. The selection criteria included professional expertise (a minimum of 10 years of experience in their respective fields), relevant backgrounds, and institutional diversity. The final panel included public health nutrition (*n* = 6), food policy (*n* = 2), marketing (*n* = 2), clinical medicine (*n* = 1), government administration (*n* = 1), and communications (*n* = 1). This composition ensured that key stakeholder perspectives from academia, government, and the private sector were well-represented, with all experts possessing a minimum of 10 years of professional experience. This robust and experienced panel is essential for a comprehensive and credible evaluation of the policy environment.

Prior to evaluation, experts reviewed the Food-EPI methodology, evidence set, and scoring criteria. The evaluation process included a dual-focus approach: one slide outlined China’s policies, and the other compared them with international benchmarks [19]. During the workshop, each indicator was assessed based on its alignment with international best practices, using a predefined scoring table (Appendix A). Scores ranged from 1 to 5 (Table 2).

The term “global best practices” or “international benchmarks” refers to the official standards outlined in the Food-EPI manual developed by the INFORMAS research group. These benchmarks consist of a set of evidence-based policy actions and implementation examples that have been demonstrated to effectively improve food environments across various international contexts. In this study, the benchmark standards were derived from the authoritative document *“Benchmarking Food Environments 2017”* [20].

The scoring criteria in Table 2 directly link a policy’s implementation level to a numerical score. The “Adherence to Best Practices (%)” column serves as a qualitative descriptor, representing the extent to which a policy aligns with international benchmarks. For example, a score of 2 indicates that the expert panel judged a policy to have rudimentary structures, aligning with 20% to 40% of the defined best practices.

All experts scored each indicator independently, and the final score was calculated as the arithmetic mean of all ratings. As the primary aim was to obtain a consensus-based aggregate rather than measure statistical agreement, formal inter-rater reliability coefficients were not computed. Averaging reduced individual variability, while any score deviating by more than two standard deviations from the mean was re-checked against the rubric to confirm accuracy. All experts used an identical scoring guide reviewed in advance, minimizing bias and improving scoring consistency.

### 2.4. Statistical Analysis

The evaluation formula used to calculate the policy effectiveness score, as presented in Table 3, was adapted from the original Food-EPI manual and validated in previous studies using the same framework [19]. This formula provides a standardized, quantitative measure of implementation, which is useful for assessing policy effectiveness and allows for comparisons across different policy domains and against international best practices.

Based on this methodology, indicator scores were calculated as the mean of expert ratings, with implementation percentages derived from these scores. Indicator scores were calculated as the arithmetic mean of expert ratings, with implementation percentages derived as (Average Score ÷ 5 × 100%). Total scores and overall implementation percentages were computed to measure cumulative policy effectiveness. Implementation levels were categorized as high (≥75%), medium (50–75%), low (25–50%), and very low (<25%), with detailed descriptive statistics provided in the Table 3. All calculations in this study were performed using Microsoft Excel (Microsoft Office 2016; Microsoft Corporation, Redmond, WA, USA.

## 3. Results

### 3.1. Characteristics of Evidence on Government Policy on Food Retail in China

The analysis of food retail policies in China demonstrated significant variations across indicators (Table 4). A total of 16 policies were identified and analyzed. National policies accounted for 37.5% of the total policies (*n* = 6) and primarily focused on RETAIL 3 (government support for healthy food) and RETAIL 4 (healthy food promotion). In contrast, provincial policies constituted the majority of the findings, accounting for 62.5% of the total policies (*n* = 10). These policies placed a strong emphasis on RETAIL 1 (local fast-food zoning restrictions) and RETAIL 2 (healthy food access).

In terms of policy type, regulations were the predominant framework, accounting for 62.5% (*n* = 10), and covered all RETAIL 1 and most RETAIL 2 policies. Guidelines accounted for 45.0% (*n* = 9) and were specifically focused on RETAIL 4. Regarding mandatory levels, the high-level category was the most common (62.5%, *n* = 10), especially in RETAIL 1 and RETAIL 2. Medium (25.0%, *n* = 4) and low (12.5%, *n* = 2) mandatory levels were only observed in RETAIL 2 and RETAIL 4, typically linked to local pilot programs and advocacy initiatives. Detailed information on these Chinese policies and documents can be found in Appendix B. Detailed benchmarks of best practice for each indicator were found in Appendix C.

### 3.2. Expert Evaluation Workshop Conduction

The expert panel (*n* = 13) was carefully structured to represent a wide range of stakeholders. It included representatives from leading academic institutions (*n* = 7), national-level research organizations (*n* = 5), government agencies (*n* = 3), and large-scale food industry stakeholders (*n* = 2). Gender diversity was also considered, with a balanced representation of 7 males and 6 females. In addition, 10 experts who had previously contributed to the evidence pack review process were also invited to the scoring workshop, ensuring continuity and enhancing the evaluation process through their specialized knowledge.

### 3.3. Score, Implementation Percentage, and Implementation Level in Food Retail

Food retail-related policies in China demonstrated significant variations across four indicators, as detailed in Table 5. The average score for the food retail domain was 2.3 ± 1.1 (45.8% implementation rate, categorized as “Low”). The scores for the four individual indicators were 1.7 ± 0.6 (low level), 2.6 ± 1.3 (medium level), 2.3 ± 1.1 (low level), and 2.5 ± 1.0 (medium level), respectively.

### 3.4. Provincial Policies Enforce Fast-Food Zoning to Control Fast-Food Outlet Density

All policies targeting local fast-food zoning restrictions (*n* = 6) were exclusively enacted at the provincial level, with no national-level interventions identified [21,22,23,24,25,26]. These regulations demonstrated high mandatory enforceability (100%) and were consistently identified as legally enforceable regulatory instruments. These policies were implemented across many provinces, such as Heilongjiang, Guangdong, Anhui, Shaanxi, Beijing, and Gansu. Detailed information regarding evidence on local fast-food zoning restrictions in China is provided below (Table 6).

### 3.5. Multi-Tiered Policies Improve Healthy Food Access in Retail Outlets

Policies governing healthy food access were primarily developed at the provincial level (80%, *n* = 4). Both tiers demonstrated strong enforcement levels, with notable differences in their scopes. National policies (20%, *n* = 1) emphasized macro-level guidance, such as stabilizing vegetable production and supply chains [27], while provincial regulations addressed operational specifics, including community vegetable delivery systems, supermarket renovations, and convenience store development [28,29,30,31]. This multi-tiered approach effectively combines broad strategic direction with localized implementation strategies (Table 7).

### 3.6. National-Level Support Enhances Healthy Food Access in Schools

The policies under RETAIL 3 (government support for healthy food) (*n* = 2) were administered exclusively at the national level, focusing on institutional food environments. Key initiatives included school food safety protocols and rural student nutrition programs, both characterized by high mandatory enforceability [32,33]. The centralized implementation of these regulations reflects the national prioritization of child nutrition and food safety (Table 8).

### 3.7. Voluntary Initiatives Promote Healthy Food in Service Environments

Three initiatives were launched to promote healthy food service environments (Table 9) [34,35,36]. These included two targeted action plans and one voluntary guideline. The action plans were designed to reduce sodium and fat intake through public education campaigns, with the goal of promoting healthier dietary choices [34]. Meanwhile, the voluntary guideline encouraged restaurants to adopt operational standards that prioritize health and wellness. In contrast with other retail indicators that typically employ mandatory frameworks, promoting healthy food in services was primarily driven by voluntary measures [35,36].

## 4. Discussion

China’s food retail policies are implemented through a hybrid governance model that combines centralized policy directives with considerable autonomy at the provincial level. While China has made progress in healthy food access and promotion, areas such as equitable fast-food zoning and transparency in institutional food governance remain in need of further development. To our knowledge, this is the first study to systematically assess China’s food retail policies in the context of global best practices, providing a standardized framework for evaluating policy implementation across key domains. By applying the Food-EPI framework in a large, rapidly developing country with a unique policy context, this study generates valuable data to inform future research and offers globally relevant insights. It also provides evidence-based policy recommendations aimed at mitigating diet-related noncommunicable diseases and promoting public health equity in China.

In the domain of fast-food zoning (RETAIL 1), for example, provincial-level laws governing restrictions on the density and location of fast-food restaurants demonstrated robust enforcement capacity. However, this decentralized approach resulted in inconsistent regional restrictions, addressing the critical need for national coordination to enhance policy effectiveness. These variations were possibly due to urban–rural disparities and the higher density of fast-food outlets near schools in urban areas, which correlates with increased childhood obesity risk [37]. This stands in stark contrast to the equity-driven models observed in nations like New Zealand [38] and Argentina [39], where zoning regulations are explicitly linked to deprivation indices or income-stratified urban policies to address health disparities rooted in socioeconomic status. Similarly, Malta’s spatially equitable controls on confectionery density offer a valuable model for China to address its significant geographic inequities more systematically [40].

In the domain of healthy food access (RETAIL 2), China’s policies, which include initiatives for fresh produce distribution and food supply stability, have achieved moderate implementation, positioning the country ahead of several high-income and middle-income countries like Germany [20], Japan [41], and Mexico [15]. This relative strength aligns with the proactive stance of Singapore in promoting healthier food environments [42]. However, despite outperforming Japan’s fresh produce distribution systems [41], China’s approach neglects key elements, such as in-store marketing regulation, a critical gap given Australia’s evidence linking shelf placement to dietary outcomes [43]. While China matches Singapore in terms of general food availability [42], both countries lag behind New Zealand in adopting nutrient-density-based shelf-length standards, which ensure more equitable visibility and access to healthy foods [44].

A similar pattern emerges in institutional food governance (RETAIL 3) and food service promotion (RETAIL 4). China’s centralized school nutrition programs (RETAIL 3), which prioritize child health, reflect international best practices like the U.S. National School Lunch Program. However, they lack transparency and participatory monitoring mechanisms, such as New Zealand’s FoodBack framework [45], a limitation also shared by Germany [20], Japan [41], Mexico [15], and Poland [46]. In contrast to these centralized directives, food service promotion (RETAIL 4) has historically relied on voluntary guidelines. This reliance is largely due to the food service industry’s market dynamics and profit motives, where unhealthy foods often generate higher margins, leading businesses to resist policies promoting healthier options. Moreover, low consumer awareness and demand for healthy food services, combined with regional disparities in economic development and resource availability, reduce incentives for enterprises to actively support such initiatives, thereby limiting policy effectiveness [37]. This approach limits its potential impact, especially when contrasted with New Zealand’s binding standards, such as junk-food-free checkout policies [44]. While Canada and the European Union lacked specific regulatory measures in this domain [47,48], China still maintains a stronger policy presence in this domain than Germany [20], Japan [20], Mexico [15], and the European Union [48], positioning it as a mid-level performer in global comparisons.

## 5. Conclusions

Overall, our study revealed that China’s food retail policy system exhibits disparities in implementation efficacy, characterized by robust provincial-level performance in areas such as food access and service promotion but presents insufficient national government involvement in zoning regulations and institutional support. These discrepancies may be attributed to vast regional disparities in governance capacity and economic development.

Beyond its findings, this study contributes theoretically and methodologically to the global discourse on food environment governance. It highlights how local adaptation within a centralized system drives healthy food policy progress, reflecting China’s unique cultural and institutional context. Methodologically, it demonstrates the Food-EPI framework’s flexibility across diverse settings, offering a standardized tool to identify implementation gaps and prioritize actions at multiple administrative levels. By benchmarking China’s food retail policies against global best practices, the study enables policymakers, researchers, and public health stakeholders to understand policy strengths and weaknesses and target interventions effectively.

To address the identified gaps in implementation, several operational strategies are recommended:

(1) Integrate socioeconomic metrics into zoning regulations, inspired by New Zealand’s deprivation-index-linked approach [38], and adopt Argentina’s income-stratified urban retail policies [39]. Establish a national legal framework to enforce uniform policies, particularly in rural regions with limited infrastructure.
(2) Transition to binding standards, as seen in New Zealand’s junk-food-free checkout initiatives [44], and implement participatory monitoring systems like New Zealand’s FoodBack framework [45]. Provide financial incentives, such as subsidies and tax breaks, to businesses promoting healthy food, especially in less developed regions.
(3) Adopt national metrics for healthy food availability, such as New Zealand’s shelf-length ratio standards [49], and expand school nutrition programs to regulate food outlets near schools. Engage communities, schools, and the media to foster a cultural shift towards healthier eating habits.
(4) Launch pilot programs and stakeholder workshops to co-develop tailored interventions and enhance multi-sector collaboration, strengthen capacity building and resource allocation in underserved areas, and improve monitoring and adaptive governance mechanisms to ensure equitable and effective policy implementation.

Despite these strengths, the study is limited by its reliance on policy documents and cross-country comparisons, which may overlook local implementation nuances. Future research should incorporate local-level data and stakeholder perspectives to refine policy recommendations. Additionally, longitudinal studies are needed to assess the long-term impact of food retail policies on dietary behaviors and health outcomes, ensuring evidence-based and adaptive governance.

## Figures and Tables

**Table 1:** Food Retail Indicators and Contextual Descriptions [15,18,19].

Indicators	Description
RETAIL 1 (Local fast-food zoning restrictions)	Zoning laws and policies are robust to empower local governments to enforce restrictions on the density and location of fast-food restaurants and other retailers selling unhealthy foods.
RETAIL 2 (Healthy food access in outlets)	Zoning laws and policies are strong enough to ensure the provision of healthy options, such as fruits and vegetables, in food service establishments.
RETAIL 3 (Government support for healthy food)	The government ensures that existing support systems are implemented to enhance the availability of healthy foods and restrict the availability of unhealthy foods in retail settings.
RETAIL 4 (Promote healthy food in services)	The government ensures that the current support system is operational to promote the availability and supply of healthy foods and to limit the promotion and supply of unhealthy foods in food service outlets.

**Table 2:** Scoring Criteria for Policy Alignment with Best Practices.

Description	Adherence to Best Practices (%) ^1^	Score
Absence or nominal policy presence	<20%	1
Rudimentary policy structures	20–40%	2
Intermediate policy implementation	40–60%	3
Robust policy strategies	60–80%	4
Comprehensive policy framework	>80%	5

^1^ The “Adherence to Best Practices” column indicates the extent to which domestic policies align with recognized international best practices.

**Table 3:** Formulas for Indicator and Total Score Calculation [19].

Step	Description	Formula
**1. Indicator Score Determination (IS** **)**	The average score for each food retail policy indicator, calculated from expert evaluations. Scores range from 0 to 5 points.	$\mathrm{IS}\;n=\frac{\sum \left(\mathrm{Expert}\;\mathrm{Scores}\right)}{\mathrm{Number}\;\mathrm{of}\;\mathrm{Experts}}$
**2. Indicator Implementation Percentage (Indicator Impl %** **)**	The percentage representing the implementation level for each indicator’s policy, relative to the maximum possible score (5 points), reflecting adherence to international benchmarks.	$\mathrm{Indicator}\;\mathrm{Impl}\;\%\;n=\left(\frac{\mathrm{Indicator}\;\mathrm{Score}\;n}{5}\right)\times 100\%$
**3. Total Score (TS)**	The overall average score across all four food retail policy indicators.	$\mathrm{TS}=\frac{\sum_{i=1}^{4}\mathrm{Indicator}\;\mathrm{Score}\;i}{4}$
**4. Total Implementation Percentage (Total Impl %)**	The overall percentage of policy implementation across all four indicators, relative to the maximum possible total score (5 points).	$\mathrm{Total}\;\mathrm{Impl}\;\%\;n=\left(\frac{\mathrm{Total}\;\mathrm{Score}\;n}{5}\right)\times 100\%$

IS*n*: Indicator Score for indicator *n*; *∑*: Summation symbol; “Expert Scores”: Scores given by individual experts; “Number of Experts”: Total count of experts; TS: Total Score; IS*i*: Indicator Score for indicator *i*.

**Table 4:** Distribution of Food Retail Policies by Governance Hierarchy and Regulatory Modalities in China.

Dimension of Policy		*n*	Proportion (%)
Level	National	6	37.5
	Provincial	10	62.5
Type	Regulations	10	62.5
	Guideline	6	37.5
Mandatory	High	10	62.5
	Medium	4	25
	Low	2	12.5

Note: The proportion (%) for each dimension was calculated based on a total of 16 identified policies. For example, the proportion of national policies (37.5%) was derived from the calculation (*n* = 6/Total *n* = 16) × 100%.

**Table 5:** Distribution of Food Retail Policies by Key Dimensions in China.

Domain	Average Score	The Level of Food Retail Policies Implementation	Low Level	Medium Level	High Level
Food retail overall	2.3 ± 1.1	45.8%	Low		
RETAIL 1 (Local fast-food zoning restrictions)	1.7 ± 0.6	33.8%	Low		
RETAIL 2 (Healthy food access in outlets)	2.6 ± 1.3	52.4%	Medium		
RETAIL 3 (Government support for healthy food)	2.3 ± 1.1	46.2%	Low		
RETAIL 4 (Promote healthy food in services)	2.5 ± 1.0	50.8%	Medium		

Note: Scores and implementation percentages were calculated based on the methodology detailed in the Statistical Analysis section. For each indicator, experts rated policies on a 0–5 scale using a standardized scoring tool. The Indicator Score is the mean of all expert ratings, calculated as the sum of all expert scores divided by the number of experts. The Implementation Percentage is derived by dividing the Indicator Score by the maximum possible score (5 points) and multiplying by 100%. Implementation levels were categorized as “High” (≥75%), “Medium” (50–75%), “Low” (25–50%), and “Very Low” (<25%). For example, RETAIL 1 (restrictive zoning for fast-food outlets) received a mean expert score of 1.7, corresponding to an Implementation Percentage of 33.8% (1.7/5 × 100%), which is classified as low implementation.

**Table 6:** Evidence on Local Fast-Food Zoning Restrictions in China (*n* = 6).

File Type	Document	Agency	Date of Issue	Target Population	Content
Regulations (National)	No documents identified				
Regulations (Province)	*Heilongjiang Province’s Food Safety Rule*	Heilongjiang Provincial Government	October 2019	Food stalls near schools	Prohibits food stalls within a 200 m radius of kindergartens and primary and secondary schools [26]
	*Guangdong Province’s Regulation on the Management of Food Production and Processing Workshops and Food Stalls*	Guangdong Provincial Government	1 October 2015	Food stalls near schools	Mandates restrictions on food vendor activities near kindergartens and schools [23]
	*Anhui Province Food Safety Rules*	Anhui Provincial Government	December 2017	Food stalls near schools	Restricts food vendors near educational institutions [22]
	*Regulations on the Administration of Small Food Processing Factories, Small Restaurants, and Food Stalls in Shaanxi Province*	Shaanxi Provincial Government	30 July 2015	Food vendors near schools	Restricts food vendors near educational institutions [25]
	*Beijing Night Market Food Service Food Safety Supervision and Management Regulation*	Beijing Municipal Government (implemented by district or county governments)	1 April 2013	Night market catering activities	Requires night market catering activities to be in designated areas, at least 200 m away from primary/secondary schools and childcare institutions [21]
Regulations (Notices)	*Province-wide Notice Prohibiting “Spicy Strips” Sales near Schools*	Gansu Provincial Market Supervision Bureau	16 March 2019	Food vendors near schools	Prohibits “spicy strips” sales within 200 m of schools [24]

**Table 7:** Evidence on Improving Healthy Food Access in Retail Outlets in China (*n* = 5).

File Type	Document	Agency	Date of Issue	Target Population	Content
Regulations (National)	*Notice of the State Council on Further Promoting Vegetable Production and Guaranteeing the Basic Stability of Market Supply and Prices*	State Council of China	27 August 2010	City mayors and urban vegetable markets	Emphasize the critical role of city mayors in managing urban vegetable markets and call for improvements in the construction, services, and management of wholesale and retail vegetable markets [27]
Regulations (Province)	*Guidance on Strengthening the Management of Community Vegetable Drive-Through Vehicles*	Beijing Municipal Bureau of Commerce	16 January 2019	Operators and managers of community vegetable drive-through vehicles	Standardize the operation and management of community vegetable drive-through vehicles, detailing the roles of relevant departments, the recruitment of businesses, and the supervision of operations [30]
	*Promotion of Fresh Supermarkets to Transform the Implementation of the Farmer Market Policy*	Xiamen Municipal Government	17 January 2007	Fresh supermarkets	Require fresh supermarkets to maintain timely stock replenishment and adjust product variety to meet consumer needs [28]
	*Convenience Stores (Community Fresh Produce Direct Sales Points) Program*	Guiyang Municipal Government	7 March 2012	Fresh produce direct sales points and hypermarkets	Mandate that fresh produce direct sales points offer at least 40 types of fresh vegetables and agricultural products, with hypermarkets required to provide at least 60 different types [31]
	*Specifications for the Establishment and Management of Fresh Food Supermarkets*	Beijing Municipal Government	12 March 2025	General merchandise stores, community food retail settings, and fresh supermarkets	Specify that fresh food sales must constitute at least 60% of total sales and detail zoning requirements for various food types [29]

**Table 8:** Evidence on Enhancing Healthy Food Access in Schools in China (*n* = 2).

File Type	Document	Agency	Date of Issue	Target Population	Content
Regulations (National)	*Regulations on School Food Safety and Nutrition Health Management*	Ministry of Education, State Administration of Market Supervision and Administration, and National Health Commission	1 April 2019	Canteens, supermarkets, and other food stores operating within kindergartens, primary, and secondary schools	Prohibits the operation of canteens, supermarkets, and other food stores within kindergartens, primary, and secondary schools, except with legal permission. Even with permission, the sale of high-salt, high-sugar, and high-fat foods is strictly prohibited [33]
	*Rural Compulsory Education Student Nutrition Improvement Plan Implementation Measures*	Ministry of Education (China)	23 May 2012	Schools participating in the rural compulsory education nutrition program	Specifies that food provided must meet food safety and nutrition health standards and respect the dietary habits of ethnic minorities. Encourages the use of fresh, high-nutrient foods and the avoidance of high-salt, high-oil, and high-sugar foods. Promotes local procurement of agricultural products to reduce costs and ensure freshness [32]

**Table 9:** Evidence on Promoting Healthy Food in Service Environments in China (*n* = 3).

File Type	Document	Agency	Date of Issue	Target Population	Content
Action Plan (National)	*Action on Salt China (ASC) Initiative*	Action on Salt China (ASC)	28 March 2017	Restaurants	Engages customers, wait staff, and chefs through educational materials and training programs [34]
Guideline (National)	*Three Reductions and Three Health (TRTH) Initiative*	Chinese Government (State Council)	22 January 2017	General population	Promote the reduction in salt, oil, and sugar intake while enhancing oral health, healthy weight, and bone health through nationwide public health campaigns [35]
Guideline (National)	*Nutritional Health Restaurant Construction Guidelines*	National Health Commission	25 December 2020	Foodservice operators	Guide foodservice operators to create nutritious and health-focused dining environments through actionable recommendations [36]

## Data Availability

All relevant data are presented in the manuscript. No supplementary datasets are available, as all necessary information is contained herein.

## Abbreviations

The following abbreviations are used in this manuscript:

BMI	Body mass index
Food-EPI	Healthy Food Environment Policy Index
INFORMAS	International Network for Food and Obesity/NCDs Research, Monitoring and Action Support
NCDs	Noncommunicable diseases
UNICEF	United Nations International Children’s Emergency Fund

## Appendix A

Food Retail Policies in China: The Healthy Food Environment Policy Index Evaluation Rating Scale

**Name**	
**Index Content**	**Indicator 1:** Zoning laws and policies are robust to empower local governments to enforce restrictions on the density and location of fast-food restaurants and other retailers selling unhealthy foods.
**International Best Practices**	**South Korea:** Since 2019, South Korea has implemented the “Green Food Zones” initiative under the Special Act on Children’s Dietary Life Safety Management [50]. This policy prohibits the sale of high-calorie, low-nutrient foods, such as candies, ice cream, carbonated beverages, and burgers, within a 200 m radius of schools. By 2016, over 10,000 schools had been included in these zones. **Detroit (USA):** Detroit’s zoning ordinance, established in 1998, bans fast-food restaurants within 500 feet of primary, secondary, and high schools [51]. **United Kingdom:** In the UK, approximately fifteen local authorities have adopted supplemental planning documents to regulate the proximity of hot food takeaways to sensitive areas, particularly schools. For instance, in 2009, the London Borough of Waltham Forest introduced a policy prohibiting hot food takeaways within 400 m of schools, parks, and youth centers. Similarly, in 2010, the London Borough of Barking and Dagenham extended this restriction to areas within a 10 min walk from schools. Comparable policies were subsequently adopted by the councils of St. Helens in 2011 and Halton in 2012 [51].
**Local Evidence**	
**Regulations (Province)**	The implementation of *Heilongjiang Province’s Food Safety Rules* established a 200 m radius around kindergartens and primary and secondary schools, where food stalls are prohibited, on October 1, 2019 [26]. Similarly, the *Guangdong Province Food Production and Processing Workshops and Food Stall Regulations* also ban food vendor activities in areas surrounding kindergartens and schools [23]. Other regions, such as Shaanxi Province and Anhui Province, have enacted similar regulations [22,25]. On March 16, 2019, the Gansu Provincial Market Supervision Administration issued a notice prohibiting the sale of “spicy strips”—a high-fat, high-salt, and additive-laden instant noodle product—within 200 m of all school campuses across the province [24]. The notice highlights the health risks associated with frequent consumption of these products and encourages citizens to report illegal production, sale, or food safety concerns related to “spicy strips.” Similar rules were adopted by Qinghai Province, Zhangzhou in Fujian Province, and other regions [52,53]. Additionally, the *Beijing Night Market Food Service Food Safety Supervision and Management Regulations*, effective 1 April 2013, require that night market catering activities be conducted in government-designated areas located at least 200 m away from the entrances of primary and secondary schools, as well as childcare institutions [21].
**Rating Score (Full points is 5)**	Given the importance of food environments in shaping dietary behaviors and diet-related health outcomes, this study evaluated China’s food retail policies through international benchmarking to identify implementation gaps and propose evidence-based strategies for improving dietary environments and public health.
**Suggestion**	(*To enhance the performance of this indicator, what specific recommendations do you propose for the future content and execution of related policies?*)

## Appendix B

Policies and Documents for Four Food Retail Indicators in China

**Food Retail Indicator**	**Name**	**Type**	**Agency**	**Date of Issue**	**Mandatory Level**
**RETAIL 1** **(Local fast-food zoning restrictions)**	Heilongjiang Province’s Food Safety Rules [26]	Regulations (Provincial)	Heilongjiang Provincial Government	1 October 2019	High
	Regulation on the Management of Guangdong Province Food Production and Processing Workshops and Food Stalls [23]	Regulations (Province)	Guangdong Provincial Government	1 October 2015	High
	Food Safety Rules (Anhui Province) [22]	Regulations (Province)	Anhui Provincial Government	1 December 2017	High
	Regulations on the Administration of Small Food Processing Factories, Small Restaurants, and Food Stalls (Shaanxi Province) [25]	Regulations (Province)	Shaanxi Provincial Government	30 July 2015	High
	Beijing Night Market Food Service Food Safety Supervision and Management Measures [21]	Regulations (Province)	Beijing Municipal Government	1 April 2013	High
	Gansu Provincial Market Supervision and Administration Bureau’s Announcement on Spicy Strips [24] (also, Qinghai Province, Zhangzhou in Fujian Province, and others) [52,53]	Guidance (Province)	Gansu Provincial Market Supervision and Administration Bureau	16 March 2019	High
**RETAIL 2** **(Healthy food access in outlets)**	Notice of the State Council on Further Promoting Vegetable Production and Guaranteeing the Basic Stability of Market Supply and Prices [27]	Guidance (National)	Chinese State Council	27 August 2010	High
	Guidance on Strengthening the Management of Community Vegetable Drive-Through Vehicle [30]	Guidance (Province)	Beijing Municipal Bureau of Commerce	16 January 2019	Medium
	Xiamen Government on the Promotion of Fresh Supermarkets to Transform the Implementation of the Farmer Market [28]	Guidance (Province)	Xiamen Government	2007	Medium
	Guiyang Construction of the Convenience Stores (Community Fresh Produce Direct Sales Points) Program 2012 [31]	Guidance (Province)	Guiyang Government	7 March 2012	Medium
	Specifications for the Establishment and Management of Fresh Food Supermarkets [29]	Regulation (Province)	Beijing Government	12 March 2025	High
**RETAIL 3** **(Government support for healthy food)**	Regulations on School Food Safety and Nutrition Health Management [33]	Regulation (National)	Ministry of Education, State Administration of Market Supervision and Administration, National Health Commission	1 April 2019	High
	Rural Compulsory Education Student Nutrition Improvement Plan Implementation Measures [32]	Guidance (National)	Ministry of Education, National Development and Reform Commission, Ministry of Finance, Ministry of Agriculture and Rural Affairs, National Health Commission, State Administration for Market Regulation, National Disease Control Bureau	31 October 2022	High
**RETAIL 4** **(Promote healthy food in services)**	Action On Salt China (ASC) [34]	Guidance (National)	George Institute for Global Health Queen Mary University of London China Center for Disease Control and Prevention China Center for Health Education National Center for Food Safety Risk Assessment	2017	Low
	Three Reduction and Three Health (TRTH) [36]	Guidance (National)	Chinese Government (as part of the Medium- and Long-term Plan for Prevention and Treatment of Chronic Diseases)	2017	Medium
	Nutritional Health Restaurant Construction Guidelines [35]	Guidance (National)	Various Local Borough Councils	December 2020	Low

## Appendix C

International Best Practices for Food Retail Domain Four Indicators

**Food Retail Indicator**	**Name**	**Type**	**Agency**	**Date of Issue**	**Mandatory Level**
**RETAIL 1** **(Local fast-food zoning restrictions)**	Green Food Zones (South Korea) [50]	Law (National)	South Korean Government	2019	High
	Zoning Ordinance (Detroit, USA) [51]	Regulations (State)	Detroit Local Government	1998	High
	Local Borough Council Planning Policies (UK) [51]	Regulations (National)	Various Local Borough Councils	2009, 2010, 2011, 2012	High
**RETAIL 2** **(Healthy food access in outlets)**	Healthy Food Financing Initiative (USA) [51]	Regulations (Underprivileged areas)	US Congress	February 2014	Medium
	New Jersey Food Access Initiative (USA) [51]	Regulations (State)	New Jersey Local Government	2016	Medium
	New York City Green Cart Permit (USA) [51]	Regulations (National)	New York City Government	2008	Medium
	Food Retail Expansion to Support Health Program (FRESH) (New York City, USA) [54]	Regulations (National)	New York City Government	2009	Medium
	Healthy Living Neighborhood Shops (Scotland) [55]	Regulations (National)	Scottish Executive	2004	Medium
**RETAIL 3** **(Government support for healthy food)**	Special Supplemental Nutrition Program for Women, Infants, and Children (WIC) (USA) [56]	Regulations (National)	US Department of Agriculture	2009	High
**RETAIL 4** **(Promote healthy food in services)**	Healthier Dining Program (Singapore) [57]	Regulations (National)	Health Promotion Board	2014	High
	Health Food Incentives Ordinance (San Francisco, USA) [58]	Regulations (State)	San Francisco Local Government	2012	High
	Ordinance No. NS300-820 (Santa Clara County, USA) [51]	Law (State)	Santa Clara County Local Government	2012	High
	Sweetened Beverage Ban (France) [51]	Law (National)	French Government	2017	High
